# Association of *MTHFR* C677T and A1298C Polymorphisms with Ovarian Reserve in Infertile Women with Thyroid Autoimmunity

**DOI:** 10.3390/biomedicines14092003

**Published:** 2026-09-06

**Authors:** Tina Sušanj Šepić, Neda Smiljan Severinski, Jadranka Vraneković, Sanja Dević Pavlić

**Affiliations:** 1 Department of Human Reproduction, Clinic for Gynecology and Obstetrics, University Hospital Centre, 51000 Rijeka, Croatia; tina.susanj@gmail.com (T.S.Š.); nedass@medri.uniri.hr (N.S.S.); 2 Department of Medical Biology and Genetics, Faculty of Medicine, University of Rijeka, 51000 Rijeka, Croatia; jadranka.vranekovic@uniri.hr

**Keywords:** assisted reproductive technology, autoimmune thyroiditis, infertility, methylenetetrahydrofolate reductase, single nucleotide polymorphism

## Abstract

**Background/Objectives**: Variability in the methylenetetrahydrofolate reductase (*MTHFR*) gene has been implicated in reproductive dysfunction and may influence ovarian physiology in women with thyroid autoimmunity (TAI). This prospective study aimed to investigate the association of the *MTHFR* C677T and A1298C polymorphisms with ovarian reserve and controlled ovarian hyperstimulation (COH) outcomes in infertile women with TAI. **Methods:** Genomic DNA was extracted from peripheral blood samples of 145 euthyroid infertile women with TAI undergoing assisted reproductive treatment and 100 fertile controls. The TAI cohort was divided into women with preserved ovarian reserve (TAI1) and diminished ovarian reserve (TAI2). *MTHFR* C677T and A1298C polymorphisms were genotyped using polymerase chain reaction–restriction fragment length polymorphism (PCR-RFLP) analysis. Genotype and allele frequencies were compared between groups, and associations with COH outcomes were evaluated. **Results**: Significant differences in *MTHFR* C677T genotype and allele distributions were observed between the TAI1 and TAI2 subgroups. Genotype distributions also differed significantly between the overall TAI cohort and fertile controls, with significant differences observed between the TAI1 subgroup and controls, but not between the TAI2 subgroup and controls. No significant associations were found for the *MTHFR* A1298C polymorphism, while associations between *MTHFR* polymorphisms and COH outcomes were limited and inconsistent. **Conclusions**: The *MTHFR* C677T, but not A1298C, polymorphism is associated with ovarian reserve in infertile women with TAI. These findings suggest that genetic variation in *MTHFR* may contribute to the heterogeneity of ovarian reserve in this population, while its influence on ovarian response to stimulation appears limited.

## 1. Introduction

Thyroid autoimmunity (TAI), characterized by the presence of thyroid autoantibodies with or without thyroid dysfunction, is the most common autoimmune condition affecting women of reproductive age [1,2]. Beyond its established association with thyroid disease, TAI has increasingly been linked to impaired fertility and adverse reproductive outcomes, even in euthyroid women [1,2,3,4,5]. In women undergoing infertility treatment, thyroid function is routinely assessed and, when necessary, stabilized before medically assisted reproduction. In women with TAI, particularly those with Hashimoto’s thyroiditis, reproductive dysfunction may reflect both the presence of thyroid autoimmunity and alterations in thyroid function. However, euthyroid women with persistent thyroid autoantibodies may still experience reproductive abnormalities despite normal circulating thyroid hormone levels. Although the relationship between TAI and reproductive dysfunction is well recognized, the underlying mechanisms remain incompletely understood. Several hypotheses have been proposed, including direct effects of thyroid autoantibodies on ovarian tissue and the reproductive tract, local thyroid hormone deficiency within the ovary, and broader immune dysregulation leading to impaired folliculogenesis, oocyte maturation, implantation, and early embryonic development [1,2,3,6,7,8,9,10]. However, these mechanisms have not been conclusively established, and the factors underlying the heterogeneous reproductive outcomes observed among women with TAI remain unclear.

Several studies have reported reduced ovarian reserve, diminished response to controlled ovarian hyperstimulation (COH), abnormalities in oocyte maturation, and less favorable outcomes following assisted reproductive technologies (ART) in women with TAI [9,11,12,13,14,15,16]. However, these findings have not been consistently replicated, suggesting that additional factors, including genetic susceptibility, may contribute to interindividual differences in ovarian reserve and subsequent response to assisted reproductive treatment in women with thyroid autoimmunity.

One candidate genetic factor is the methylenetetrahydrofolate reductase (*MTHFR*) gene, which encodes a key enzyme in folate metabolism that catalyzes the conversion of 5,10-methylenetetrahydrofolate to 5-methyltetrahydrofolate, the methyl donor required for different methylation reactions in the cell [17,18]. This pathway is essential for DNA synthesis and epigenetic regulation, processes that are critical for normal folliculogenesis, oogenesis, and early embryonic development. Among the numerous *MTHFR* variants identified, the common single-nucleotide polymorphisms (SNPs) C677T (rs1801133) and A1298C (rs1801131) reduce enzymatic activity to varying degrees and may consequently alter folate metabolism and DNA methylation [19].

Because epigenetic regulation is involved in both immune homeostasis and reproductive function, *MTHFR* polymorphisms have been investigated as potential contributors to autoimmune diseases and infertility [20,21,22]. Altered *MTHFR* activity has been suggested to impair DNA methylation and immune regulation and has been associated with reproductive processes, including follicular development, oocyte competence, embryo quality, implantation, and response to ovarian stimulation [21,23,24]. In addition, *MTHFR* polymorphisms have been suggested to influence susceptibility to autoimmune diseases through alterations in methylation-dependent regulation of immune responses [23]. However, the biological mechanisms underlying these associations remain incompletely understood.

Numerous studies have investigated the relationship between *MTHFR* polymorphisms and reproductive outcomes, particularly in women undergoing ART [25,26,27,28]. Several reports have associated the C677T polymorphism with altered ovarian reserve, impaired oocyte maturation, poorer ovarian response to stimulation, reduced embryo quality, implantation failure, and lower cumulative live birth rates. Likewise, the A1298C variant and combined C677T/A1298C genotypes have been associated with differences in ovarian response and embryological outcomes [29,30]. However, other studies have found no significant associations between *MTHFR* polymorphisms and ART success [21,26,31,32]. These inconsistent findings likely reflect differences in study design, study populations, ethnic background, folate status, and outcome measures.

Importantly, few studies have specifically examined the relationship between *MTHFR* polymorphisms and ovarian function in women with TAI [25,32,33]. Since thyroid autoimmunity and altered MTHFR activity may independently influence immune regulation, methylation pathways, and ovarian physiology, their coexistence may contribute to the heterogeneity of reproductive outcomes observed in this population. However, the association between *MTHFR* polymorphisms and ovarian reserve in women with TAI remains poorly understood.

Therefore, the aim of the present study was to investigate the association between the *MTHFR* C677T and A1298C polymorphisms and ovarian reserve in infertile women with thyroid autoimmunity, and to further evaluate whether these polymorphisms are associated with response to controlled ovarian hyperstimulation during assisted reproductive treatment.

## 2. Materials and Methods

### 2.1. Study Population

This study included 145 infertile women with TAI who underwent COH and ART at the Department of Human Reproduction, Clinical Hospital Centre Rijeka, Croatia, between 2018 and 2023.

A control group consisting of 100 fertile women was included for comparison of *MTHFR* genotype frequencies. The control women were recruited during the same period (2018–2023) from women of reproductive age who had conceived naturally and delivered at least one healthy child without the use of ART. The control group had a normal BMI (18.5–24.9 kg/m^2^), and none of the control participants had a history of thyroid disease.

Women younger than 38 years with confirmed infertility and TAI were eligible for inclusion in the study group. All patients underwent a standard infertility evaluation including medical and gynecological history, physical examination, assessment of reproductive hormone levels, thyroid function, and thyroid autoantibodies. Thyroid autoimmunity was defined as the presence of positive thyroid peroxidase antibodies (TPOAbs) and/or thyroglobulin antibodies (TgAbs). In the present cohort, thyroid autoimmunity corresponded to Hashimoto’s thyroiditis, and all included women were euthyroid at the time of study inclusion, with TSH values within the reference range.

Exclusion criteria for the TAI group included the presence of autoimmune diseases other than thyroid autoimmunity that could potentially affect fertility, as well as previously diagnosed polycystic ovary syndrome (PCOS), endometriosis, and age > 38 years. Women in the control group were required to meet the same exclusion criteria, except for infertility and thyroid autoimmunity.

Ovarian reserve was assessed only in women with TAI using serum anti-Müllerian hormone (AMH) concentrations [34]. Women with an AMH concentration ≥ 1.1 ng/mL were classified as having preserved ovarian reserve (TAI1, *n* = 91), whereas those with an AMH concentration < 1.1 ng/mL were classified as having diminished ovarian reserve (TAI2, *n* = 54). Serum follicle-stimulating hormone (FSH), luteinizing hormone (LH), estradiol (E2), thyroid-stimulating hormone (TSH), thyroid peroxidase antibodies (TPOAbs), and thyroglobulin antibodies (TgAbs) concentrations were also determined as part of the routine endocrine evaluation.

The study was conducted in accordance with the principles of the Declaration of Helsinki and was approved by the Ethics Committee of the Faculty of Medicine, University of Rijeka, and the Clinical Hospital Centre Rijeka (Approval No. 007-06/23-02/244 (27 December 2022) and 2170-29-02/1-22-2 (19 August 2022), respectively). Written informed consent was obtained from all participants prior to enrollment.

### 2.2. Controlled Ovarian Hyperstimulation

All women in the TAI cohort underwent controlled ovarian hyperstimulation using a flexible gonadotropin-releasing hormone (GnRH) antagonist protocol followed by transvaginal ultrasound-guided oocyte retrieval. Fertilization was performed by either conventional in vitro fertilization (IVF) or intracytoplasmic sperm injection (ICSI), according to semen characteristics. Embryological assessment was performed according to the Vienna Consensus criteria, while blastocyst morphology was evaluated using the Gardner grading system.

The evaluated COH outcomes included the total number of retrieved oocytes, proportion of metaphase II (MII) oocytes, fertilization rate, cleavage rate (day 3 embryos), and blastocyst rate.

### 2.3. Hormonal Measurements

Serum concentrations of FSH, LH, E2, and AMH were measured using electrochemiluminescence immunoassays on the Cobas^®^ 6000 e601 analyzer (Roche Diagnostics, Mannheim, Germany), while serum TSH, TPOAbs, and TgAbs concentrations were determined using chemiluminescent immunoassays on the Atellica^®^ IM1600 analyzer (Siemens Healthineers, Dublin, Ireland), all according to the manufacturer’s instructions.

### 2.4. DNA Isolation and MTHFR Genotyping

Peripheral venous blood samples (3–5 mL) were collected from all participants for genetic analysis. Genomic DNA was isolated from peripheral blood leukocytes using the FlexiGene DNA Kit (Qiagen GmbH, Hilden, Germany) according to the manufacturer’s protocol. DNA concentration and purity were assessed spectrophotometrically before analysis.

Genotyping of the *MTHFR* C677T (rs1801133) and A1298C (rs1801131) polymorphisms was performed using a standard polymerase chain reaction followed by restriction fragment length polymorphism (PCR-RFLP) protocol [35]. PCR amplification was carried out using a Mastercycler^®^ Personal thermal cycler (Eppendorf, Hamburg, Germany). Restriction enzyme digestion was performed using enzymes obtained from New England Biolabs (Ipswich, MA, USA) according to the manufacturer’s instructions. PCR products and restriction fragments were separated by electrophoresis on 3% agarose gels stained with GelRed™ (Olerup SSP^®^, Saltsjöbaden, Sweden) and visualized under ultraviolet illumination.

### 2.5. Statistical Analysis

Statistical analyses were performed using R software and Statistica for Windows, version 13.3 (StatSoft Inc., Tulsa, OK, USA).

Continuous variables are presented as mean ± standard deviation (SD) or median (interquartile range), as appropriate, while categorical variables are presented as counts and percentages. Genotype distributions were tested for conformity with Hardy–Weinberg equilibrium using the chi-square goodness-of-fit test. Differences in genotype and allele frequencies between women with preserved and diminished ovarian reserve were analyzed using the chi-square (χ^2^) test. Associations between *MTHFR* polymorphisms and ovarian reserve, as well as differences in controlled ovarian hyperstimulation outcomes across genotypes, were evaluated using linear regression models with genotype included as a categorical predictor. All statistical tests were two-sided, and *p* < 0.05 was considered statistically significant.

## 3. Results

### 3.1. Study Population Characteristics

A total of 145 infertile women with thyroid autoimmunity (TAI) were included in the study. Of these, 91 women were classified as TAI1 (preserved ovarian reserve) and 54 as TAI2 (diminished ovarian reserve) based on serum AMH concentrations. All participants in the TAI cohort were of reproductive age and had a normal BMI (18.5–24.9 kg/m^2^). Baseline clinical, hormonal, and immunological characteristics of the TAI study population are presented in Table 1.

In addition, a control group comprising 100 fertile women of reproductive age with a normal BMI, a history of natural conception, and at least one live birth was included for comparison of *MTHFR* genotype frequencies. The mean age of the control group was 30.16 ± 5.18 years.

### 3.2. Distribution of MTHFR Genotypes

Both *MTHFR* C677T and A1298C genotype distributions were in Hardy–Weinberg equilibrium in the TAI and control groups. The distribution of *MTHFR* C677T genotype and allele frequencies according to ovarian reserve among women with thyroid autoimmunity is presented in Table 2. The distribution of *MTHFR* C677T genotypes differed significantly between women with preserved ovarian reserve (TAI1) and those with diminished ovarian reserve (TAI2) (χ^2^ = 14.68, *p* < 0.001). Likewise, allele frequencies differed significantly between the two subgroups (χ^2^ = 17.10, *p* < 0.0001). The CC genotype was more frequent in women with preserved ovarian reserve than in those with diminished ovarian reserve (64.8% vs. 33.3%), whereas the CT (27.5% vs. 44.4%) and TT (7.7% vs. 22.2%) genotypes were more common in the TAI2 subgroup. Similarly, the C allele predominated in the TAI1 subgroup (78.6% vs. 55.6%), whereas the T allele was more frequent in women with diminished ovarian reserve (21.4% vs. 44.4%).

Comparison with the fertile control group (Table 3) showed a significantly different distribution of *MTHFR* C677T genotypes between women with TAI and controls (χ^2^ = 6.55, *p* = 0.038), whereas allele frequencies did not differ significantly (χ^2^ = 1.94, *p* = 0.163). When the TAI cohort was analyzed according to ovarian reserve, significant differences were observed between the TAI1 subgroup and fertile controls in both genotype distribution (χ^2^ = 12.91, *p* = 0.002) and allele frequencies (χ^2^ = 9.81, *p* = 0.002), whereas no significant differences were observed between the TAI2 subgroup and fertile controls for either genotype distribution (χ^2^ = 3.49, *p* = 0.175) or allele frequencies (χ^2^ = 2.10, *p* = 0.147).

No significant differences in *MTHFR* A1298C genotype or allele frequencies were observed between women with preserved and diminished ovarian reserve (Table 4) (genotypes: χ^2^ = 0.28, *p* = 0.867; alleles: χ^2^ = 0.26, *p* = 0.612). Comparison with fertile controls (Table 5) likewise revealed no significant differences between fertile controls and women with TAI (Table 5) (genotypes: χ^2^ = 4.35, *p* = 0.114; alleles: χ^2^ = 3.57, *p* = 0.059).

The frequency of compound heterozygous *MTHFR* C677T/A1298C (CT/AC) carriers was 26.0% in fertile controls, 13.8% in the overall TAI group, 8.8% in the TAI1 subgroup, and 22.2% in the TAI2 subgroup. Significant differences were observed between fertile controls and the overall TAI group (χ^2^ = 5.78, *p* = 0.016), between the TAI1 and TAI2 subgroups (χ^2^ = 5.14, *p* = 0.023), and between fertile controls and the TAI1 subgroup (χ^2^ = 9.64, *p* = 0.002), whereas no significant difference was observed between fertile controls and the TAI2 subgroup (χ^2^ = 0.27, *p* = 0.604).

### 3.3. Association Between MTHFR Polymorphisms and Controlled Ovarian Hyperstimulation Outcomes in Women with Thyroid Autoimmunity

Because controlled ovarian hyperstimulation (COH) outcomes were available only for infertile women with thyroid autoimmunity, subsequent analyses were performed exclusively within the TAI cohort. Regression analyses were conducted separately in the TAI1 and TAI2 subgroups to evaluate the association between *MTHFR* polymorphisms and COH outcomes.

In the TAI1 group, carriers of the *MTHFR* C677T CT genotype had a significantly higher cleavage rate than women with the CC genotype (β = 14.82, 95% CI 1.09–28.55, *p* = 0.035). In addition, women carrying the *MTHFR* A1298C CC genotype had a significantly higher proportion of metaphase II (MII) oocytes than those with the AA genotype (β = 19.01, 95% CI 3.81–34.21, *p* = 0.015). No significant associations were observed between *MTHFR* polymorphisms and the remaining evaluated COH outcomes, including the total number of retrieved oocytes, fertilization rate, and blastocyst development.

In the TAI2 group, neither the C677T nor the A1298C polymorphism was significantly associated with any evaluated COH outcome.

The complete regression analyses are presented in Supplementary Tables S1 and S2, with a graphical summary shown in Supplementary Figure S1.

## 4. Discussion

The present study identified the *MTHFR* C677T polymorphism as a genetic factor associated with ovarian reserve in infertile women with thyroid autoimmunity. Women in the TAI2 subgroup exhibited a significantly higher frequency of the T allele and TT genotype than women in the TAI1 subgroup, whereas no such association was observed for the A1298C polymorphism. Furthermore, comparison with a fertile control group demonstrated significant differences in *MTHFR* C677T genotype distribution between women with thyroid autoimmunity and fertile controls. When the TAI cohort was analyzed according to ovarian reserve, significant differences were observed between the TAI1 subgroup and fertile controls, whereas no significant differences were found between the TAI2 subgroup and fertile controls. Together, these findings suggest that the relationship between *MTHFR* C677T and A1298C polymorphisms, thyroid autoimmunity, and ovarian reserve is more complex than a simple association with infertility or thyroid autoimmunity alone. Rather, genetic variation in one-carbon metabolism may contribute to the heterogeneity of ovarian reserve among women with thyroid autoimmunity.

Comparison with the fertile control group further highlighted the complexity of these associations. Although genotype distributions differed significantly between the overall TAI cohort and fertile women, this difference was largely attributable to the TAI1 subgroup, whereas the TAI2 subgroup showed genotype frequencies comparable with those observed in fertile controls. This unexpected pattern may indicate that the *MTHFR* C677T variant does not simply increase susceptibility to infertility or thyroid autoimmunity, but may instead modify ovarian susceptibility to metabolic and immune-related factors in genetically predisposed women [2,3,9]. One possible explanation is that, among women with preserved ovarian reserve, the effect of *MTHFR* C677T may be more apparent, whereas in women who already have diminished ovarian reserve, the ovarian phenotype may reflect the combined effects of multiple genetic, metabolic, and autoimmune factors, thereby attenuating the apparent contribution of a single polymorphism. However, this interpretation remains speculative, particularly given the relatively small size of the TAI1 (*n* = 91) and TAI2 (*n* = 54) subgroups. The observed pattern may therefore represent a signal of a more complex interaction between MTHFR genotype, ovarian reserve, and thyroid autoimmunity, but it cannot be considered conclusive [26,28,36]. These findings should be further evaluated in larger, well-characterized cohorts with detailed assessment of autoimmune, metabolic, and genetic factors.

The observed association between the *MTHFR* C677T polymorphism and diminished ovarian reserve is biologically consistent with the established role of MTHFR in one-carbon metabolism [25,26,27,28]. MTHFR catalyzes the conversion of 5,10-methylenetetrahydrofolate into 5-methyltetrahydrofolate, the principal methyl donor required for homocysteine remethylation and numerous methylation reactions involved in DNA synthesis, repair, and epigenetic regulation [18]. These processes are essential for folliculogenesis, granulosa cell proliferation, oocyte maturation, and early embryonic development [25,26,27,28]. The C677T substitution substantially reduces MTHFR enzymatic activity, particularly in TT homozygotes, resulting in impaired folate metabolism, elevated homocysteine concentrations, oxidative stress, and altered DNA methylation [18,35,36]. These disturbances have all been proposed as mechanisms contributing to accelerated follicular depletion and impaired ovarian function. In women with thyroid autoimmunity, such metabolic alterations may further interact with chronic immune activation, local inflammatory responses, and altered ovarian immune homeostasis, thereby increasing susceptibility to diminished ovarian reserve [20,37].

Thyroid autoimmunity itself has been associated with chronic low-grade inflammation, altered follicular immune homeostasis, and impaired ovarian reserve, although the underlying mechanisms remain incompletely understood [1,2]. Because MTHFR activity influences one-carbon metabolism and DNA methylation, processes involved in both immune regulation and ovarian function, genetic variation in the *MTHFR* gene may further modify the reproductive phenotype of women with TAI [25,26,27,28,38,39]. The present findings support this concept by demonstrating that not all women with thyroid autoimmunity exhibit the same genetic profile in relation to ovarian reserve.

Compared with C677T, the A1298C polymorphism was not associated with ovarian reserve in women with thyroid autoimmunity, either in comparisons between the TAI1 and TAI2 subgroups or between women with thyroid autoimmunity and fertile controls. This finding is consistent with the lower functional impact of the A1298C variant on MTHFR activity [30]. Unlike the C677T substitution, which markedly reduces enzymatic activity and is frequently associated with elevated homocysteine concentrations, the A1298C variant generally produces a more modest reduction in enzyme function and, when present alone, usually has little effect on circulating homocysteine levels or folate metabolism [30,40]. Consequently, its influence on DNA methylation and ovarian physiology is expected to be less pronounced. Although some investigators have reported associations between the *MTHFR* A1298C polymorphism and markers of ovarian reserve or ovarian responsiveness, including higher basal FSH concentrations or altered AMH levels, most studies evaluating assisted reproductive outcomes have found no independent association with ovarian reserve, embryo development, or pregnancy outcomes [21]. Our findings therefore support the notion that the C677T polymorphism represents the functionally more relevant *MTHFR* variant with respect to ovarian reserve, whereas A1298C, at least in isolation, appears to exert little influence on ovarian function in women with thyroid autoimmunity.

The analysis of compound heterozygous *MTHFR* C677T/A1298C genotypes provided additional insight into the observed genotype distribution. Compound heterozygous carriers were significantly less frequent in the overall TAI cohort than among fertile controls, with the difference primarily attributable to the TAI1 subgroup, whereas no significant difference was observed between the TAI2 subgroup and fertile controls. In addition, the frequency of compound heterozygotes was significantly higher in women with diminished ovarian reserve than in those with preserved ovarian reserve. Previous studies have suggested that compound heterozygosity may reduce MTHFR enzymatic activity to a level approaching that observed in C677T homozygotes, potentially resulting in impaired folate metabolism and altered methylation capacity [28,29,33,41]. However, because the A1298C polymorphism alone was not associated with ovarian reserve in the present study, the observed differences in compound heterozygote frequencies are more likely to reflect the contribution of the C677T variant than a true synergistic effect of both polymorphisms. Nevertheless, the biological significance of this finding remains uncertain and warrants further investigation in larger cohorts.

The influence of *MTHFR* polymorphisms on controlled ovarian hyperstimulation outcomes appeared considerably more limited than their association with ovarian reserve. Among women with preserved ovarian reserve (TAI1), carriers of the C677T CT genotype had a higher cleavage rate, while carriers of the A1298C CC genotype exhibited a higher proportion of metaphase II oocytes. However, neither polymorphism was associated with the remaining evaluated embryological parameters, including the total number of retrieved oocytes, fertilization rate, or blastocyst development, and no significant associations were observed in women with diminished ovarian reserve (TAI2). These isolated findings should therefore be interpreted with caution and do not support a consistent influence of *MTHFR* polymorphisms on ovarian stimulation outcomes in women with thyroid autoimmunity. Although age and BMI are also recognized factors that may influence COH outcomes, age did not differ significantly between the TAI1 and TAI2 subgroups (Table 1), while all women in the TAI cohort had a normal BMI (18.5–24.9 kg/m^2^), limiting the assessment of their influence on COH outcomes.

The available literature similarly reports inconsistent associations between *MTHFR* polymorphisms and assisted reproductive outcomes [23,24,31,42]. While several studies have associated the C677T TT genotype with reduced ovarian responsiveness, impaired oocyte maturation, poorer embryo quality, and lower cumulative live birth rates, others have found no significant associations or even reported more favorable embryological outcomes among carriers of the T allele [21,32,33,38,41,43,44]. Recent systematic reviews and meta-analyses likewise conclude that the overall effect of *MTHFR* polymorphisms on ART outcomes remains modest and highly dependent on study population, folate status, stimulation protocol, and outcome definition [26,28,36]. Collectively, these findings suggest that the reproductive consequences of altered MTHFR activity are context dependent and are likely modified by interactions between genetic background, folate metabolism, immune status, and other environmental factors. Within this framework, our results indicate that, in women with thyroid autoimmunity, *MTHFR* C677T appears to have greater relevance for ovarian reserve than for ovarian response during assisted reproductive treatment. The inclusion of a fertile control group and the simultaneous evaluation of both common functional *MTHFR* polymorphisms and compound heterozygous genotypes provide a more comprehensive assessment of the relationship between *MTHFR* genetic variation and ovarian reserve in women with thyroid autoimmunity.

Several limitations should be considered when interpreting these findings. The relatively modest sample size may have limited the statistical power to detect smaller associations. In addition, folate and homocysteine concentrations were not measured, limiting assessment of the potential functional effects of the *MTHFR* variants and their contribution to the observed association with ovarian reserve. Finally, the study was conducted in a single ethnic population, which may limit the generalizability of the findings. Future studies with larger and more diverse populations, including folate and homocysteine measurements, are warranted.

## 5. Conclusions

In conclusion, our findings suggest that the *MTHFR* C677T polymorphism, but not A1298C, is associated with ovarian reserve in infertile women with thyroid autoimmunity. The associations between *MTHFR* polymorphisms and controlled ovarian hyperstimulation outcomes were limited and inconsistent, suggesting that *MTHFR* C677T may be more relevant to ovarian reserve than to ovarian response to stimulation. Further studies in larger, well-characterized populations incorporating functional biomarkers of folate metabolism are needed to confirm these findings and clarify their potential clinical significance.

## Figures and Tables

**Table 1:** Baseline characteristics of infertile women with thyroid autoimmunity according to ovarian reserve.

Variable	TAI1 Mean ± SD (*n* = 91)	TAI2 Mean ± SD (*n* = 54)	*p*
Age (years)	34.96 ± 3.15	35.87 ± 2.50	0.138
AMH (ng/mL)	3.18 ± 1.48	0.665 ± 0.349	<0.001 *
FSH (IU/L)	7.28 ± 2.03	9.77 ± 4.72	<0.001 *
LH (IU/L)	6.63 ± 3.01	6.06 ± 2.77	0.255
E2 (pmol/L)	171.53 ± 94.74	203.53 ± 209.56	0.310
TSH (mIU/L)	2.32 ± 1.26	2.15 ± 1.27	0.424
TPOAbs (IU/mL)	572.93 ± 825.97	438.08 ± 566.00	0.251
TgAbs (IU/mL)	112.69 ± 191.21	166.10 ± 245.00	0.182

TAI1—women with thyroid autoimmunity and preserved ovarian reserve; TAI2—women with thyroid autoimmunity and diminished ovarian reserve; AMH—anti-Müllerian hormone; FSH—follicle-stimulating hormone; LH—luteinizing hormone; E2—estradiol; TSH—thyroid-stimulating hormone; TPOAbs—thyroid peroxidase antibodies; TgAbs—thyroglobulin antibodies. * Statistically significant (*p* < 0.05).

**Table 2:** Distribution of *MTHFR* C677T genotypes and alleles according to ovarian reserve in women with thyroid autoimmunity.

Genotype/Allele	TAI1 *n* (%)	TAI2 *n* (%)	χ^2^ Test	*p*
CC	59 (64.8)	18 (33.3)		
CT	25 (27.5)	24 (44.4)		
TT	7 (7.7)	12 (22.2)	14.68	0.00065 *
C allele	143 (78.6)	60 (55.6)		
T allele	39 (21.4)	48 (44.4)	17.10	0.000036 *

* Statistically significant (*p* < 0.05). TAI1—women with thyroid autoimmunity and preserved ovarian reserve; TAI2—women with thyroid autoimmunity and diminished ovarian reserve.

**Table 3:** Distribution of *MTHFR* C677T genotypes and alleles in fertile controls and women with thyroid autoimmunity.

Genotype/Allele	Control *n* (%)	TAI Total *n* (%)	χ^2^ Test	*p*
CC	39 (39.0)	77 (53.1)		
CT	50 (50.0)	49 (33.8)		
TT	11 (11.0)	19 (13.1)	6.55	0.038 *
C allele	128 (64.0)	203 (70.0)		
T allele	72 (36.0)	87 (30.0)	1.94	0.163

* Statistically significant (*p* < 0.05). TAI total—women with thyroid autoimmunity.

**Table 4:** Distribution of *MTHFR* A1298C genotypes and alleles according to ovarian reserve in women with thyroid autoimmunity.

Genotype/Allele	TAI1 *n* (%)	TAI2 *n* (%)	χ^2^ Test	*p*
AA	53 (58.2)	29 (53.7)		
AC	32 (35.2)	21 (38.9)		
CC	6 (6.6)	4 (7.4)	0.28	0.867
A allele	138 (75.8)	79 (73.1)		
C allele	44 (24.2)	29 (26.9)	0.26	0.612

TAI1—women with thyroid autoimmunity and preserved ovarian reserve; TAI2—women with thyroid autoimmunity and diminished ovarian reserve.

**Table 5:** Distribution of *MTHFR* A1298C genotypes and alleles in fertile controls and women with thyroid autoimmunity.

Genotype/Allele	Control *n* (%)	TAI Total *n* (%)	χ^2^ Test	*p*
AA	43 (43.0)	82 (56.6)		
AC	48 (48.0)	53 (36.6)		
CC	9 (9.0)	10 (6.9)	4.35	0.114
A allele	134 (67.0)	217 (74.8)		
C allele	66 (33.0)	73 (25.2)	3.57	0.059

TAI total—women with thyroid autoimmunity.

## Data Availability

The raw data supporting the conclusions of this article will be made available by the authors on request.

## Supplementary Materials

The following supporting information can be downloaded at https://www.mdpi.com/article/10.3390/biomedicines14092003/s1, Supplementary Table S1: Linear regression analysis of the association between *MTHFR* C677T polymorphism and controlled ovarian hyperstimulation (COH) outcomes in the TAI1 and TAI2 groups; Supplementary Table S2: Linear regression analysis of the association between *MTHFR* A1298C polymorphism and controlled ovarian hyperstimulation outcomes (COH) in the TAI1 and TAI2 groups. Supplementary Figure S1: Forest plot of linear regression analyses showing the associations between *MTHFR* C677T and A1298C polymorphisms and controlled ovarian hyperstimulation (COH) outcomes in infertile women with thyroid autoimmunity (TAI1 and TAI2 groups).

## Abbreviations

The following abbreviations are used in this manuscript:

AMH	Anti-Müllerian hormone
ART	Assisted reproductive technology
BMI	Body mass index
CI	Confidence interval
COH	Controlled ovarian hyperstimulation
E2	Estradiol
FSH	Follicle-stimulating hormone
GnRH	Gonadotropin-releasing hormone
LH	Luteinizing hormone
MTHFR	Methylenetetrahydrofolate reductase
PCR	Polymerase chain reaction
SD	Standard deviation
SNP	Single nucleotide polymorphism
TAI	Thyroid autoimmunity
TAI1	Thyroid autoimmunity with preserved ovarian reserve
TAI2	Thyroid autoimmunity with diminished ovarian reserve
TgAbs	Thyroglobulin antibodies
TPOAbs	Thyroid peroxidase antibodies
TSH	Thyroid-stimulating hormone
