# Supplementary material for: Association of MTHFR C677T and A1298C Polymorphisms with Ovarian Reserve in Infertile Women with Thyroid Autoimmunity
Source: Biomedicines. 2026 Sep 6;14(9):2003. doi: 10.3390/biomedicines14092003 (PMC13604727; doi:10.3390/biomedicines14092003)
Supplement: Supplementary file 1 [file biomedicines-14-02003-s001.zip › biomedicines-4496751-supplementary.pdf]

Supplementary Table S1. Linear regression analysis of the association between *MTHFR* C677T polymorphism and controlled ovarian hyperstimulation (COH) outcomes in the TAI1 and TAI2 groups.

	TAI1		TAI2	
COH outcome	CT vs CC<br>β (95% CI)<br>p	TT vs CC<br>β (95% CI)<br>p	CT vs CC<br>β (95% CI)<br>p	TT vs CC<br>β (95% CI)<br>p
Total number of oocytes	-1.54 (-3.80 - 0.73)<br>0.181	0.48 (-2.15 - 3.11)<br>0.717	-0.61 (-4.24 - 3.02)<br>0.738	-6.61 (-14.40 - 1.18)<br>0.095
MII oocyte rate (%)	-6.11 (-17.27 - 5.05)<br>0.280	-12.06 (-24.91 - 0.80)<br>0.066	4.48 (-7.43 - 16.39)<br>0.454	6.39 (-19.15 - 31.94)<br>0.617
Fertilization rate (%)	10.59 (-2.25 - 23.42)<br>0.105	4.03 (-10.48 - 18.55)<br>0.582	-5.69 (-20.23 - 8.85)<br>0.435	3.67 (-27.51 - 34.86)<br>0.814
Cleavage rate (%)	14.82 (1.09 - 28.55)<br>0.035	11.56 (-3.97 - 27.08)<br>0.143	-0.56 (-18.01 - 16.89)<br>0.949	15.41 (-22.01 - 52.83)<br>0.412
Blastocyte rate (%)	6.71 (-8.08 - 21.49)<br>0.370	3.54 (-13.19 - 20.27)<br>0.675	1.67 (-12.71 - 16.05)<br>0.817	-22.23 (-53.07 - 8.61)<br>0.154

Supplementary Table S2. Linear regression analysis of the association between *MTHFR* A1298C polymorphism and controlled ovarian hyperstimulation outcomes (COH) in the TAI1 and TAI2 groups.

	TAI1		TAI2	
COH outcome	AC vs AA<br>β (95% CI), p	CC vs AA<br>β (95% CI), p	AC vs AA<br>β (95% CI), p	CC vs AA<br>β (95% CI), p
Total number of oocytes	1.13 (-1.15 - 3.41),<br>0.328	2.17 (-0.99 - 5.32),<br>0.176	-3.09 (-6.76 - 0.57),<br>0.096	3.06 (-6.12 - 12.25),<br>0.506
MII oocyte rate (%)	2.35 (-8.63 - 13.34),<br>0.671	19.01 (3.81 - 34.21),<br>0.015	4.76 (-7.17 - 16.69),<br>0.427	20.60 (-9.28 - 50.48),<br>0.172
Fertilization rate (%)	-4.30 (-17.13 - 8.53),<br>0.507	-0.74 (-18.44 - 16.95),<br>0.934	1.47 (-13.45 - 16.38),<br>0.844	4.23 (-33.11 - 41.57),<br>0.821
Cleavage rate (%)	-6.89 (-20.73 - 6.96),<br>0.325	-6.64 (-25.74 - 12.47),<br>0.492	5.03 (-12.76 - 22.81),<br>0.573	14.42 (-30.11 - 58.94),<br>0.518
Blastocyte rate (%)	5.77 (-8.83 - 20.38),<br>0.434	4.24 (-15.91 - 24.38),<br>0.677	-6.60 (-21.08 - 7.89),<br>0.365	26.84 (-9.44 - 63.12),<br>0.144

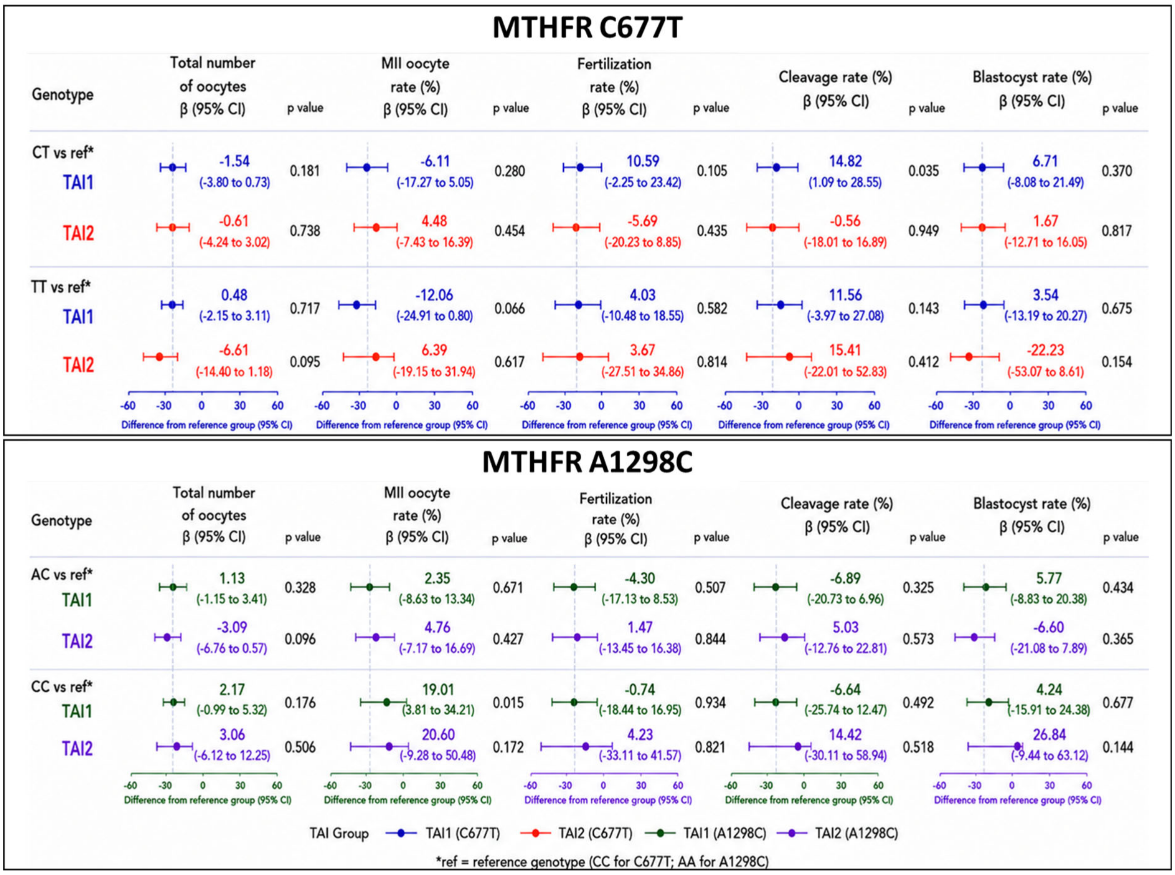

Supplementary Figure S1. Forest plot of linear regression analyses showing the associations between *MTHFR* C677T and A1298C polymorphisms and controlled ovarian hyperstimulation (COH) outcomes in infertile women with thyroid autoimmunity (TAI1 and TAI2 groups).
